# A longitudinal investigation of the effects of the COVID-19 pandemic on 2SLGBTQ+ youth experiencing homelessness

**DOI:** 10.1371/journal.pone.0288591

**Published:** 2023-07-17

**Authors:** Alex Abramovich, Nelson Pang, Kawon Victoria Kim, Rowen K. Stark, Shannon Lange, Michael Chaiton, Carmen H. Logie, Hayley A. Hamilton, Sean A. Kidd

**Affiliations:** 1 Institute for Mental Health Policy Research, Centre for Addiction and Mental Health, Toronto, Ontario, Canada; 2 Dalla Lana School of Public Health, University of Toronto, Toronto, Ontario, Canada; 3 Department of Psychiatry, University of Toronto, Toronto, Ontario, Canada; 4 Factor Inwentash Faculty of Social Work, University of Toronto, Toronto, Ontario, Canada; University of Technology Sydney, AUSTRALIA

## Abstract

**Introduction:**

The objective of this study was to examine the impacts of the coronavirus 2019 (COVID-19) pandemic on various dimensions of wellbeing among 2SLGBTQ+ youth experiencing homelessness over a 12-month period during the COVID-19 pandemic.

**Methods:**

2SLGBTQ+ youth (recruited using a convenience sampling method) participated in three online surveys to assess mental health (depression, anxiety, suicidality), substance and alcohol use, health care access, and violence for 12-months between 2021–2022. Quantitative data analysis included non-parametric one-sample proportion tests, paired t-test and McNemar’s test. Longitudinal data collected across all three timepoints were treated as paired data and compared to baseline data using non-parametric exact multinomial tests, and if significant, followed by pairwise post-hoc exact binomial tests. For the purposes of analysis, participants were grouped according to their baseline survey based on pandemic waves and public health restrictions.

**Results:**

2SLGBTQ+ youth experiencing homelessness (n = 87) reported high rates of mental health challenges, including anxiety and depression, over 12-months during the pandemic. Youth participants reported experiencing poor mental health during the early waves of the pandemic, with improvements to their mental health throughout the pandemic; however, results were not statistically significant. Likewise, participants experienced reduced access to mental health care during the early waves of the pandemic but mental health care access increased for youth throughout the pandemic.

**Conclusion:**

Study results showed high rates of mental health issues among 2SLGBTQ+ youth, but reduced access to mental health care, due to the COVID-19 pandemic. Findings highlight the need for 2SLGBTQ+ inclusive and affirming mental health care and services to address social and mental health issues that have been exacerbated by the pandemic.

## Background

The onset of the coronavirus 2019 (COVID-19) pandemic had detrimental effects on the mental health of youth, particularly increased depression and anxiety [1–3]. Existing research has demonstrated increased negative mental health impacts of the COVID-19 pandemic on structurally vulnerable youth [3–6]. Two-spirit, lesbian, gay, bisexual, transgender, queer, and questioning (2SLGBTQ+) youth, specifically, have experienced increased mental health challenges following the emergence of the COVID-19 pandemic, compared to their cisgender and heterosexual peers [7–9]. However, there has been little research on the impacts of the COVID-19 pandemic on the mental health of youth living at the intersection of 2SLGBTQ+ identity and homelessness, despite 2SLGBTQ+ youth constituting a disproportionate 20–40% of the homeless youth population in North America [10–13]. 2SLGBTQ+ youth experiencing homelessness report significantly higher rates of mental health issues compared to cisgender and heterosexual youth, largely due to discrimination, identity-based rejection and conflict, and stigma [10–12, 14]. Mental health challenges among 2SLGBTQ+ youth during the COVID-19 pandemic have been amplified by low family support, identity-based discrimination and violence, social isolation, and barriers to accessing mental health and substance use services [7, 15–18]. Some studies have also demonstrated heterogeneity within 2SLGBTQ+ youth populations, with youth experiencing multiple forms of marginalization, such as Indigenous and/or racialized 2SLGBTQ+ youth reporting particularly high rates of racism, transphobia, and homophobia, resulting in poor mental health outcomes and barriers to care [15, 19].

The limited research available suggest a range of negative impacts of the COVID-19 pandemic on 2SLGBTQ+ youth experiencing homelessness, including loss of employment, fewer safe housing options, and high rates of mental health concerns, including depression, anxiety, suicidality, self-harm, and problematic substance use [20]. Overburdened medical systems and the closure of social support programs resulted in negative consequences for 2SLGBTQ+ youth at risk of, and experiencing, homelessness. 2SLGBTQ+ youth have experienced unique stressors during the COVID-19 pandemic including being forced to isolate at home with unsupportive and abusive family members due to a lack of alternative housing options [18]

The majority of research on the impacts of the COVID-19 pandemic on youth mental health have been either cross-sectional or comparing pre-pandemic mental health to a single time point following the onset of the pandemic. Less is known about the impacts of the COVID-19 pandemic on youth mental health over time, including how different stages of the pandemic and related public health safety measures had differentially impacted mental health and well-being [21]. The existing research contains diverse findings, with some studies showing stable but poor mental health throughout the pandemic, [22] while others found fluctuations or a decrease in substance use and/or mental health concerns over the course of the pandemic [21–25]. Studies also demonstrated diverging findings regarding different stages of the pandemic and the impact of periods of heightened infection risk and lockdown measures on mental health symptoms [26–28]. Changes in youth mental health outcomes during the COVID-19 pandemic may be impacted by gender, family functioning, pre-pandemic mental and physical health, student status of youth, and exposure to pandemic-related stressors [22, 24, 27–29].

In the context of heterogeneous findings regarding the longitudinal impacts of the COVID-19 pandemic on youth mental health, it is necessary to further investigate the experiences of specific populations of youth over time.

To address these gaps, this study engaged a group of 2SLGBTQ+ youth at risk of, and experiencing, homelessness in the Greater Toronto Area (GTA) and surrounding areas in Ontario, Canada to understand their experiences of mental health and access to services over one year (2021–2022) of the COVID-19 pandemic.

## Materials and methods

This study utilizes a longitudinal mixed-methods approach to investigate the impacts associated with the COVID-19 pandemic on 2SLGBTQ+ youth at risk of, and experiencing, homelessness in the GTA and surrounding areas in Ontario, Canada. Although this was a mixed-methods study involving the collection and analysis of qualitative and quantitative data, only quantitative results will be discussed and reported in this article as the qualitative data has been reported in previous papers [18, 20, 30].

Quantitative data were collected through self-administered online surveys at three different timepoints (baseline, 3 months, 6 months). The research team assembled a Community Advisory Board (CAB) to advise on various aspects of the study, including design, survey and interview guide development, participant recruitment, and knowledge translation. Members of the CAB included frontline staff and management from youth serving organizations in the GTA and surrounding areas. This article reports findings based on data collected from January 2021 to January 2022. Research ethics approval was obtained from the Centre for Addiction and Mental Health (CAMH) Research Ethics Board (#102/2020).

### Participants

Participants were enrolled in this study on an ongoing basis between January 2021 and June 2021. Inclusion criteria for enrollment included: self-identify as 2SLGBTQ+; aged 14–29 (the Government of Canada defines youth up to 29 years);(10) at risk of, or experiencing homelessness (e.g., experiencing financial difficulties or challenges paying rent; residing at a shelter or housing program; living with family members that are unsupportive of 2SLGBTQ+ identity/lacking security or stability; living without parents/caregivers and unable to secure safe, stable, or consistent housing); living in the Greater Toronto Area (GTA) (e.g., Durham, Peel, Toronto, etc.) or surrounding areas (e.g., Waterloo, Guelph). Ninety-two youth met the inclusion criteria and were enrolled in this study.

## Quantitative methods

### Recruitment and sampling

The research team recruited 92 youth participants by utilizing a convenience sampling method and collaborating closely with youth serving organizations in the GTA and surrounding areas. The research team made use of existing relationships to network with staff from organizations in these areas. Additionally, the research team included a 2SLGBTQ+ youth peer support worker to support participant recruitment. The peer support worker recruited participants via outreach to various youth-serving organizations, advertising on social media, and conducting study information sessions at relevant organizations. Youth who were interested in participating in this study were asked to contact the research team for further details to determine eligibility. Participants enrolled in the study were compensated with an electronic gift card upon completion of each survey. A gift card of $35 was provided for completion of the baseline survey, a $40 gift card for completion of the second/follow-up survey, and a $45 gift card for completion of the third and final follow-up survey.

### Data collection

Youth who met all inclusion criteria were enrolled to participate in the study and were sent a consent form prior to the baseline survey. Written informed consent was obtained from each participant prior to the beginning of the survey. The need for parental or guardian consent was waived by the Research Ethics Board. Once the consent form was signed and all questions were answered, the participants were emailed a link to the baseline survey. Participants were informed there would be a total of three virtual surveys, the second one being sent three months after the baseline survey, and the third final survey being sent six months after the baseline survey. Survey data was anonymized after data collection. The surveys took approximately 30 minutes to complete and included a variety of questions focused on demographics, impacts of the COVID-19 pandemic on mental health, alcohol and other substance use, and health service access. Validated and standardized measures were utilized to assess anxiety (Generalized Anxiety Disorder-7 item scale [GAD-7], α = 0.927); depression (Patient Health Questionnaire [PHQ-9], α = 0.918); problematic alcohol and drug use (CAGE-AID Questionnaire), α = 0.819; and suicidality (scale derived from a four-item scale used with youth in previous studies) [31–35]. For the GAD-7, cut-off scores of 5, 10, and 15 represented mild, moderate, and severe anxiety, respectively [33]. For the PHQ-9, cut-off scores of 5, 10, 15, and 20 represented mild, moderate, moderately severe, and severe depression, respectively [32]. For the CAGE-AID screening tool, a score of two or more indicated problematic alcohol and/or substance use [34]. Health service access was measured by the following question: Has your access to mental health care changed since the start of the COVID-19 pandemic/Past 3-Months? With the responses a) No, you have not tried to access mental health care, or you haven’t needed care since March 1, 2020/Since last survey; b) No, there have been no changes to your mental health care; c) Yes, you have had mild changes, such as appointments moved online instead of in-person visits; d) Yes, you have had moderate changes, such as delays in your appointments or getting prescriptions with some impact on your mental health; e) Yes, you have had severe changes; you have been unable to access needed care with impact on your mental health. REDCap electronic data software (hosted at CAMH) was used to collect and manage survey data [36, 37]. This article shares findings based on data collected from all three surveys.

### Context

The findings shared in this article focus on data that were collected from January 2021 to January 2022. The COVID-19 pandemic began to severely impact Ontarians in March 2020, with the first provincial state of emergency declared March 17, 2020. When data collection commenced in January 2021, Ontario was facing the second wave of the pandemic. A province-wide lockdown had been imposed since December 2020 and a second provincial state of emergency was declared mid-January 2021. In February 2021, COVID-19 cases were steadily decreasing; the state of emergency was declared over and the stay-at-home order was partially lifted in certain regions of the province. The remaining regions of Ontario exited out of the stay-at-home orders in March 2021. COVID-19 cases began rising again in March and a third wave of the pandemic was declared at this time. In April 2021, Ontario reached a new peak of COVID-19 cases and a second province-wide lockdown and stay-at-home mandate was ordered, which lasted until early June 2021, when case numbers had decreased. Ontario developed a three-step plan to reopen the province and moved through all three steps in June and July 2021. Further health and safety restrictions were lifted throughout the fall of 2021 during the fourth wave of the pandemic. In late October 2021, the province released “A Plan to Safely Reopen Ontario and Manage COVID-19 for the Long-Term” which outlined a plan to lift all remaining public health measures by March 2022 [38]. This plan was not implemented as intended due to increasing case numbers throughout the winter. Data collection for this study ended shortly after the Omicron-dominated wave began in December 2021.

### Analysis

To verify the legitimacy of participants, the research team carefully reviewed participant survey responses by comparing static factors, such as age and race, between screener, baseline, and follow-up surveys. Consequently, ten participants were removed from the analysis due to significant inconsistencies in these factors across surveys. Partially completed questionnaires were included in data analysis.

Given that the survey completion dates for timepoints (T) 1, 2, and 3 differed among participants, and the varying COVID-19 public health implementations across the study period, the heterogeneity among participants was suspected to be high. Therefore, we separated participants according to the month that the T1 survey was completed and compared variables of interest across timepoints within each group. Likewise, because the pandemic cannot be viewed as simply a single event, but rather a fluid event, during which there were periods of lockdowns and restrictions, which impacted participants’ reported experiences. The groupings were done in an effort to account for this fluidity.

**Fig 1:**
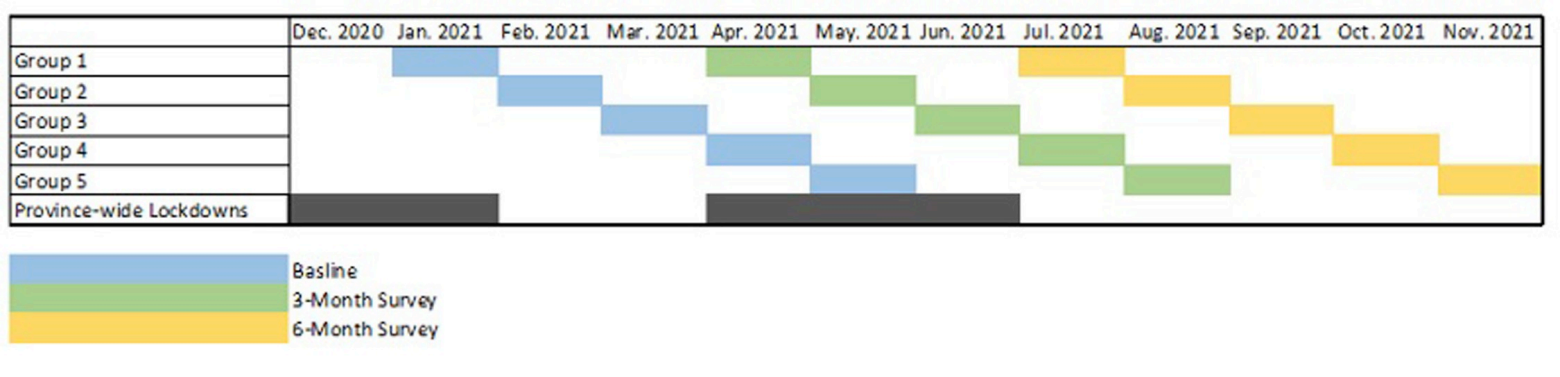
Timeline of data collection.

This resulted in five groups that respectively completed the T1 survey in January, February, March, April and May, 2021 (See Table 1 for the outline of group allocation schedule and Fig 1 for timeline of data collection). Additionally, only participants who completed the baseline survey and at least one follow-up survey were included for analysis.

**Table 1:** Outline of group allocation schedule.

Group	T1	T2	T3
**1** (n = 11)	January 2021	April 2021	July 2021
**2** (n = 14)	February 2021	May 2021	August 2021
**3** (n = 27)	March 2021	June 2021	September 2021
**4** (n = 26)	April 2021	July 2021	October 2021
**5** (n = 9)	May 2021	August 2021	November 2021

*T = Timepoint

Non-parametric tests were used for all analyses due to the small number of participants in each group and non-normally distributed data. Categorical variables that were not collected across all timepoints were assessed using a one-sample proportion test. Continuous and binary variables that were collected across all three timepoints were treated as paired data, and data from each follow up time point was compared to baseline data using the paired t-test and McNemar test, respectively. Nominal variables that were collected across all three timepoints were treated as paired data, and the exact multinomial test was performed to compare each follow up time point to baseline. If the exact multinomial test was statistically significant (*p*<0.05), a post-hoc exact binomial test was then performed pairwise. Ordinal variables that were collected across all three timepoints were treated as paired data, and the Friedman test was performed as an omnibus test to simultaneously compare all three timepoints. If the Friedman test was statistically significant (*p*<0.05), a post-hoc analysis was performed using the Wilcoxon signed rank test to compare each follow up time point to baseline. All analyses were performed in RStudio version 1.3.1073, [39] and a *p*-value less than 0.05 was considered statistically significant.

## Results

The sociodemographic characteristics of the participants are reported in Table 2. Participants had an average age of 20.22 years (SD: 3.65; Range: 15–28 years) and represented diverse ethno-racial backgrounds including Black (∼21%), Asian (∼15%), mixed-background (∼15%), and White (∼52%). Participants used a variety of terms to describe their gender identity and sexual orientation. For example, over a quarter of youth (∼26%; n = 23) identified as gender diverse and another quarter identified as cisgender women. Approximately 33% (n = 29) of youth identified as bisexual and approximately 17% (n = 15) identified as gay. There were no statistically significant differences in age, race, gender identity, or sexual orientation between the five groups.

**Table 2:** Sociodemographic characteristics of sample (n = 87).

	Number (%)	Chi-squared test (p-value)
Age Group		0.4010
16–20	55 (64%)	
21–24	16 (18.6%)	
25–29	15 (17.4%)	
**Gender Identity**		0.5565
Cisgender woman	23 (26.43%)	
Cisgender man	12 (13.79%)	
Transgender woman	11 (12.64%)	
Transgender man	14 (16.09%)	
Two-Spirit	<5	
Gender diverse (Non-Binary, Genderqueer, Genderfluid, Androgynous)	23 (26.44%)	
Unknown	<5	
**Sexual Orientation**		0.3164
Asexual	<5	
Bisexual	29 (33.33%)	
Demisexual*	<5	
Gay	15 (17.24%)	
Lesbian	14 (16.09%)	
Pansexual	11 (12.64%)	
Queer (including fluid)	13 (14.94%)	
Straight/Heterosexual	<5	
**Ethno-Racial Background**		0.8412
Asian	13 (14.94%)	
Black	18 (20.69%)	
European	17 (19.54%)	
Indigenous	<5	
Latinx	<5	
Mixed-Background	13 (14.94%)	
White	45 (51.72%)	

*Individuals who identify as “demisexual” only experience sexual attraction to someone with whom they have formed a strong emotional bond or connection with.

### Living situation

**Fig 2:**
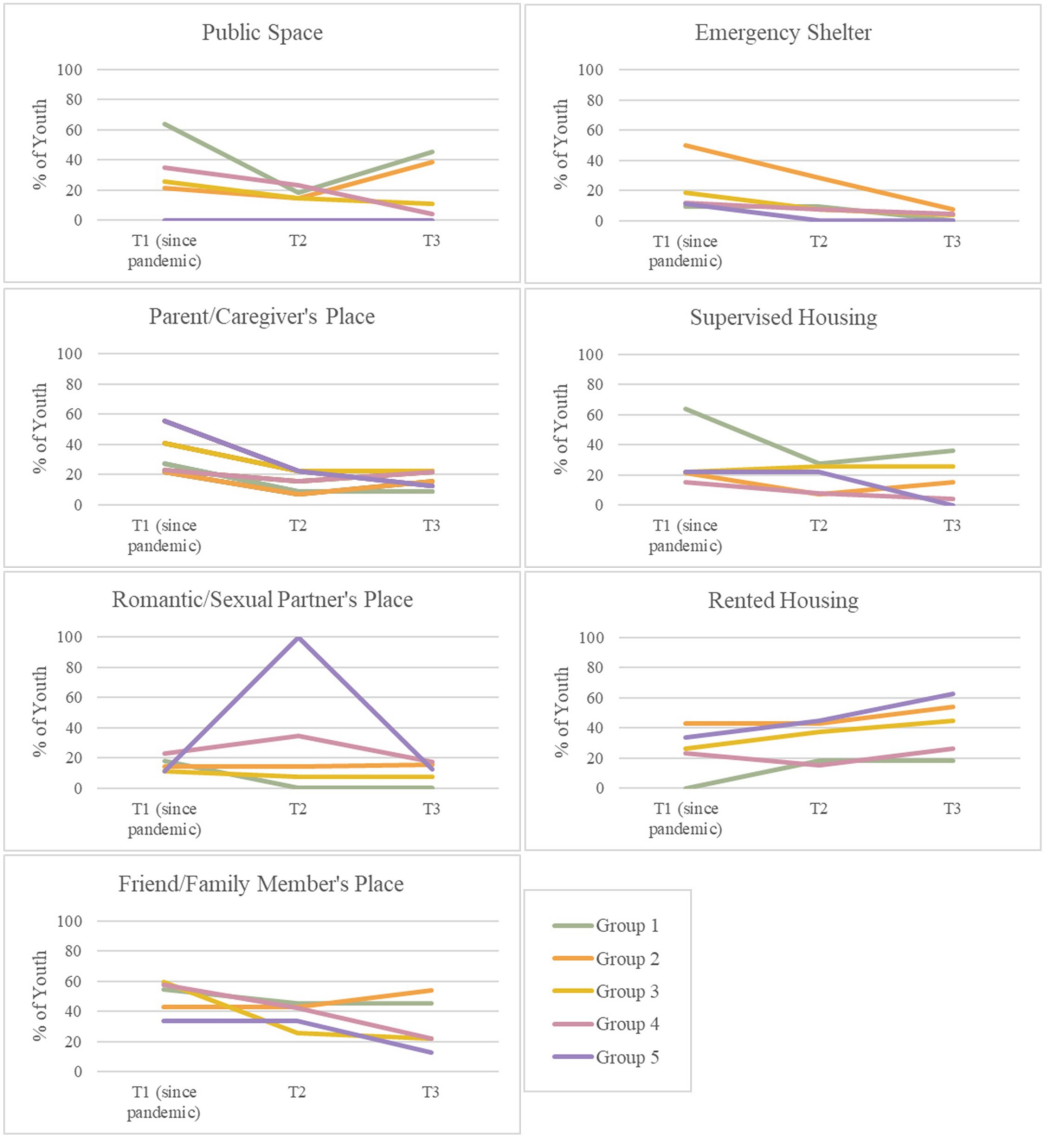
Living situations among participants throughout the pandemic.

At T1, youth were asked to indicate where they had been living since the start of the COVID-19 pandemic. At T2 and T3, youth were asked to indicate where they had been living since they last completed the survey (past 3 months). Youth were asked to report their living situations/all of the places they lived throughout the provided time period on each survey. Fig 2 illustrates how youths’ living situations changed over the course of the pandemic. In the following figures, “Public Space” includes youth who reported living in vehicles, makeshift shelters, tents or shacks, abandoned or vacant buildings, or other public spaces (e.g., sidewalks, squares, parks, forests, bus shelters); “Supervised Housing” includes youth who reported living in group homes, supervised residences, or transitional housing; and “Friend/Family Member’s Place” describes living with any friend or family who is not a parent/caregiver or romantic/sexual partner. A small number of youth (n < 5) reported living in a place they owned or a hotel/motel throughout the pandemic, thus these data and analyses are not presented.

Changes over time in the number of participants reporting each type of living situation were explored using McNemar’s Test. Comparing T1 and T2, there was a statistical difference in the number of youth living at a friend/family member’s place in Group 3 (p = 0.0159). The changes in Group 3 were characterized largely by youth who reported living at a friend/family member’s house at T1 but not T2. There were no statistical differences between T1 and T2 in the number of youth living in a place they rent, romantic/sexual partner’s place, parent/caregiver’s place, emergency shelter, supervised housing, or public space. Comparing T1 and T3, there were statistical differences in the number of youth living at a friend/family member’s place in Group 3 (p = 0.0044) and Group 4 (p = 0.0159) and differences in the number of youth living in public spaces in Group 4 (p = 0.0133). The changes in Groups 3 and 4 were characterized largely by youth who reported living at a friend/family member’s place at T1 but not T3, and youth who reported living in public spaces at T1 but not T3. There were no statistical differences between T1 and T3 in the number of youth living in a place they rent, romantic/sexual partner’s place, parent/caregiver’s place, emergency shelter, or supervised housing.

### Depression

Paired sample t-tests indicated that there were statistically significant decreases in PHQ-9 Depression scores between T1 and T2 in Group 4 [t(23) = 4.69, p < 0.001] and between T1 and T3 in Group 4 [t(18) = 3.74, p = 0.0015] and Group 5 [t(7) = 2.49, p = 0.0412]. There were no statistical differences in PHQ-9 Depression scores for the other groups. These results indicate that, across all groups, depressive symptoms either remained stable or improved throughout 2021. Table 3 displays the mean score and standard deviation for depression and anxiety scores.

**Table 3:** Changes in depression and anxiety.

	T1		T2		T3	
	Mean	SD	Mean	SD	Mean	SD
Depression (PHQ-9 Scores)						
Group 1	14	5.88	15	3.60	14.82	3.12
Group 2	15.43	7.30	13.79	6.39	14.92	6.44
Group 3	17.70	5.25	17.96	4.96	16.08	5.98
Group 4	16.04	6.77	11.17	7.69	13.05	8.13
Group 5	13.67	6.08	10.56	3.54	9.75	7.81
Anxiety (GAD-7 Scores)						
Group 1	18.91	6.39	19.81	4.67	19.36	5.37
Group 2	21	5.66	18.21	5.65	18.08	5.12
Group 3	20.70	5.12	20.56	5.65	20.54	5.78
Group 4	20.64	5.49	16.04	6.78	18.05	7.11
Group 5	18.11	4.94	16.89	2.52	14.5	6.39

**Fig 3:**
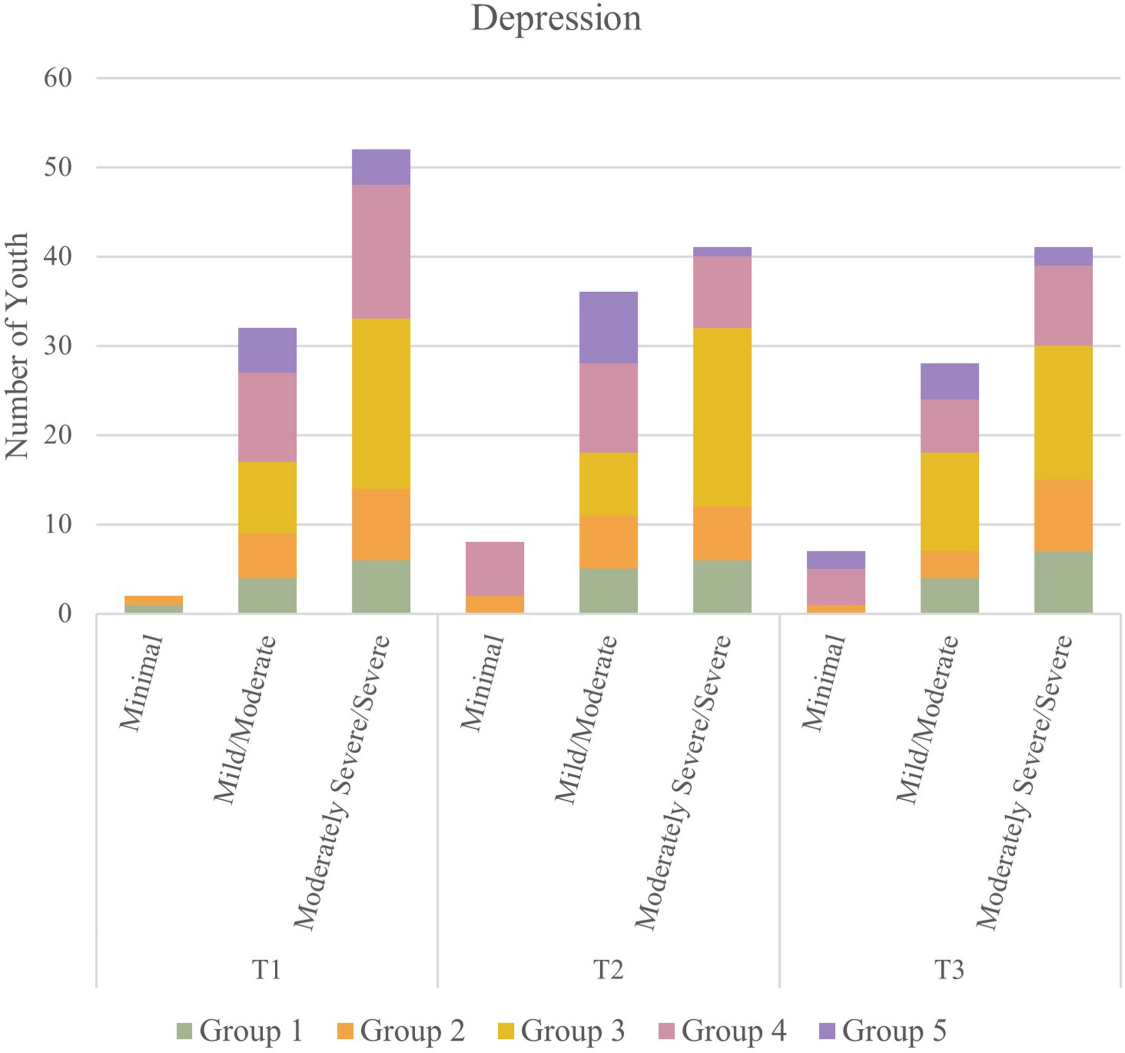
Number of 2SLGBTQ+ youth experiencing depression among participants over time during the COVID-19 pandemic.

Friedman Rank Sum tests showed that the median distribution of participants by PHQ-9 category differed significantly across timepoints in Group 4 (p = 0.0018). In this group, a post-hoc Wilcoxon signed rank test demonstrated that the distribution of participants by PHQ-9 category differed significantly between T1 and T2 (p < 0.001) and T1 and T3 (p = 0.0177). This change in distribution was characterized by youth who scored in the more severe PHQ-9 categories at T1 and in the less severe categories at T2 and T3. See Fig 3 for the categorical breakdown of depression among participants over time.

### Anxiety

**Fig 4:**
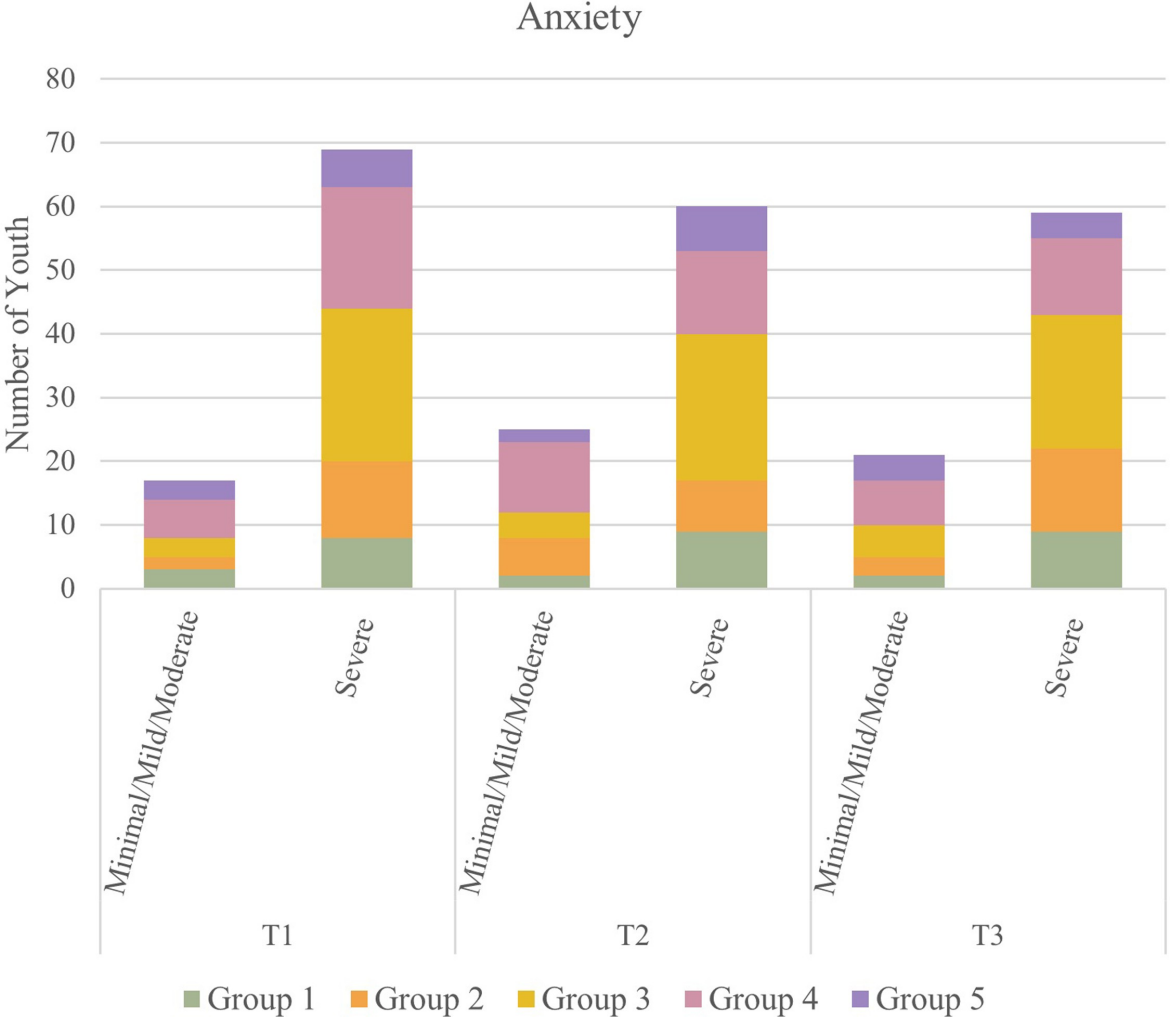
Prevalence of anxiety among participants over time.

A paired sample t-test indicated that there were statistical decreases in the GAD-7 Anxiety scores in Group 4 between T1 and T2 [t(22) = 3.99, p < 0.001] and T1 and T3 [t(18) = 2.90, p = 0.0096]. There were no statistical differences in the GAD-7 Anxiety scores in the other groups, indicating that anxiety symptoms were either stable or improved over time among all groups. McNemar’s tests showed that the distribution of participants by GAD-7 category did not differ significantly between T1 and T2 or T1 and T3 in any of the groups. See Fig 4 for categorical breakdown of anxiety among participants over time.

### Suicide & self-harm

**Fig 5:**
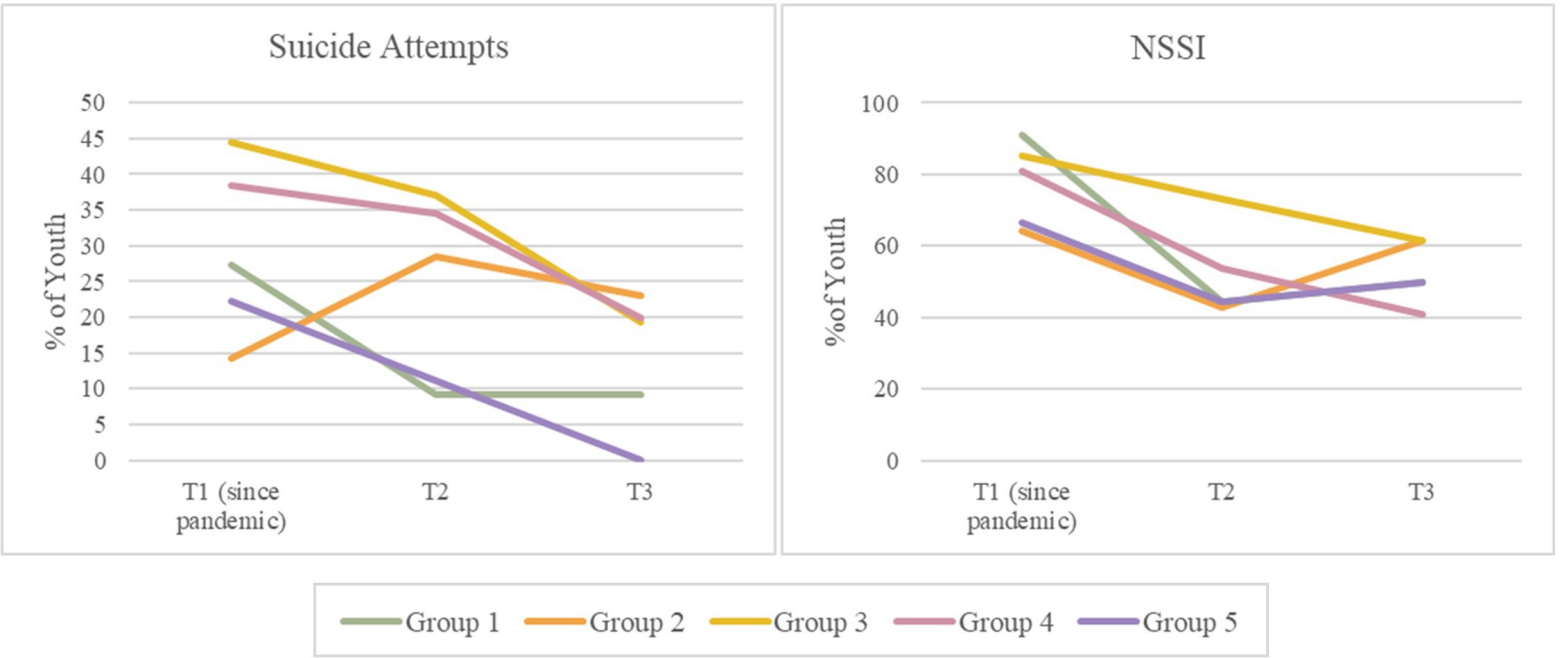
Prevalence of suicidality among participants over time.

One-sample proportions tests did not indicate any significant differences in the proportion of youth who attempted suicide (requiring medical treatment) across timepoints in any groups. One-sample proportions tests indicated a significant difference in the proportion of youth who engaged in non-suicidal self-injury (NSSI) over time in Group 4. In this group, the proportion of youth engaging in NSSI was significantly lower at T3 than at T1 (since the pandemic) or prior to the pandemic. See Fig 5 for a graphical representation of the prevalence of suicidality among participants over time.

### Substance use

**Fig 6:**
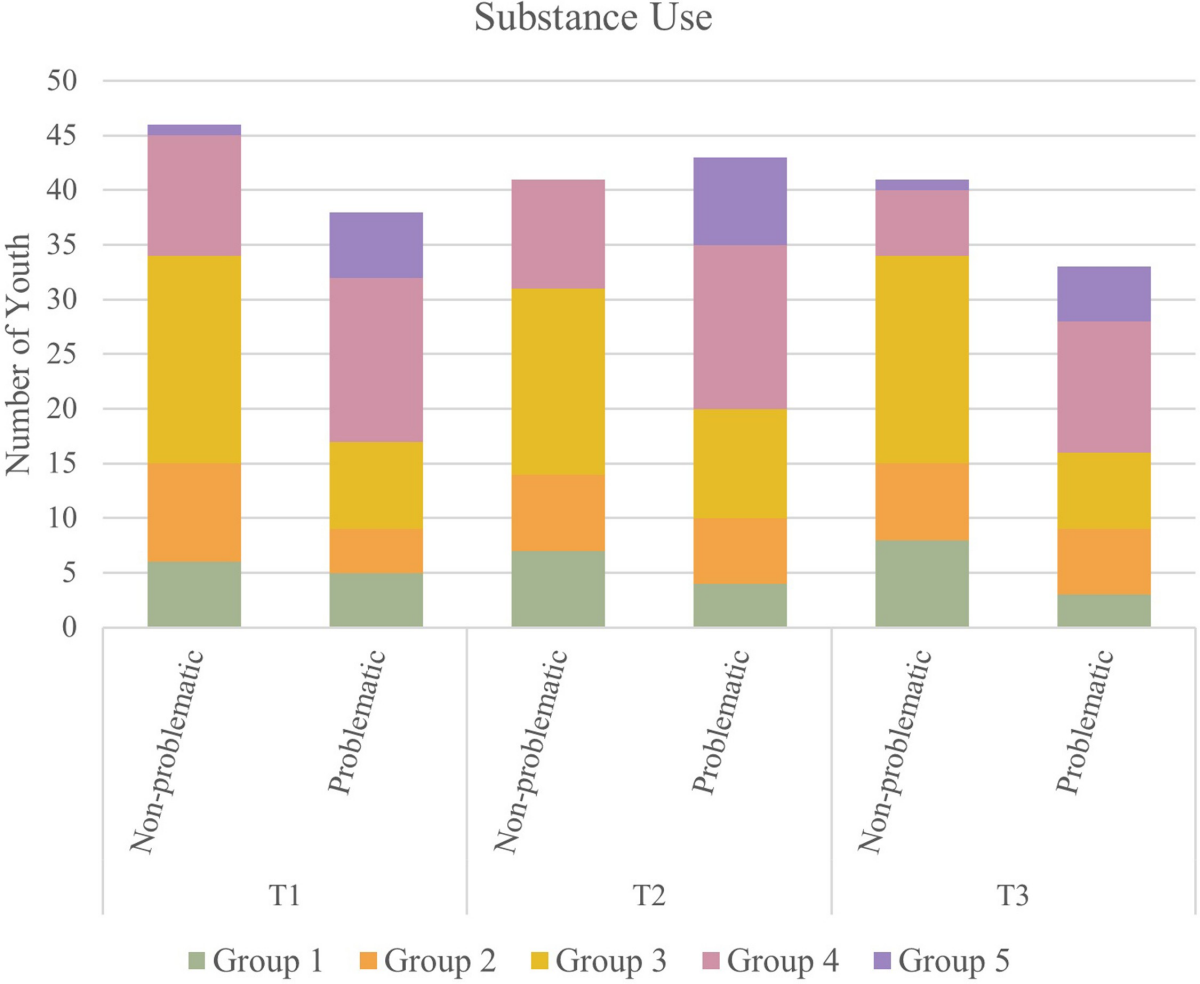
Substance use among participants over time.

**Fig 7:**
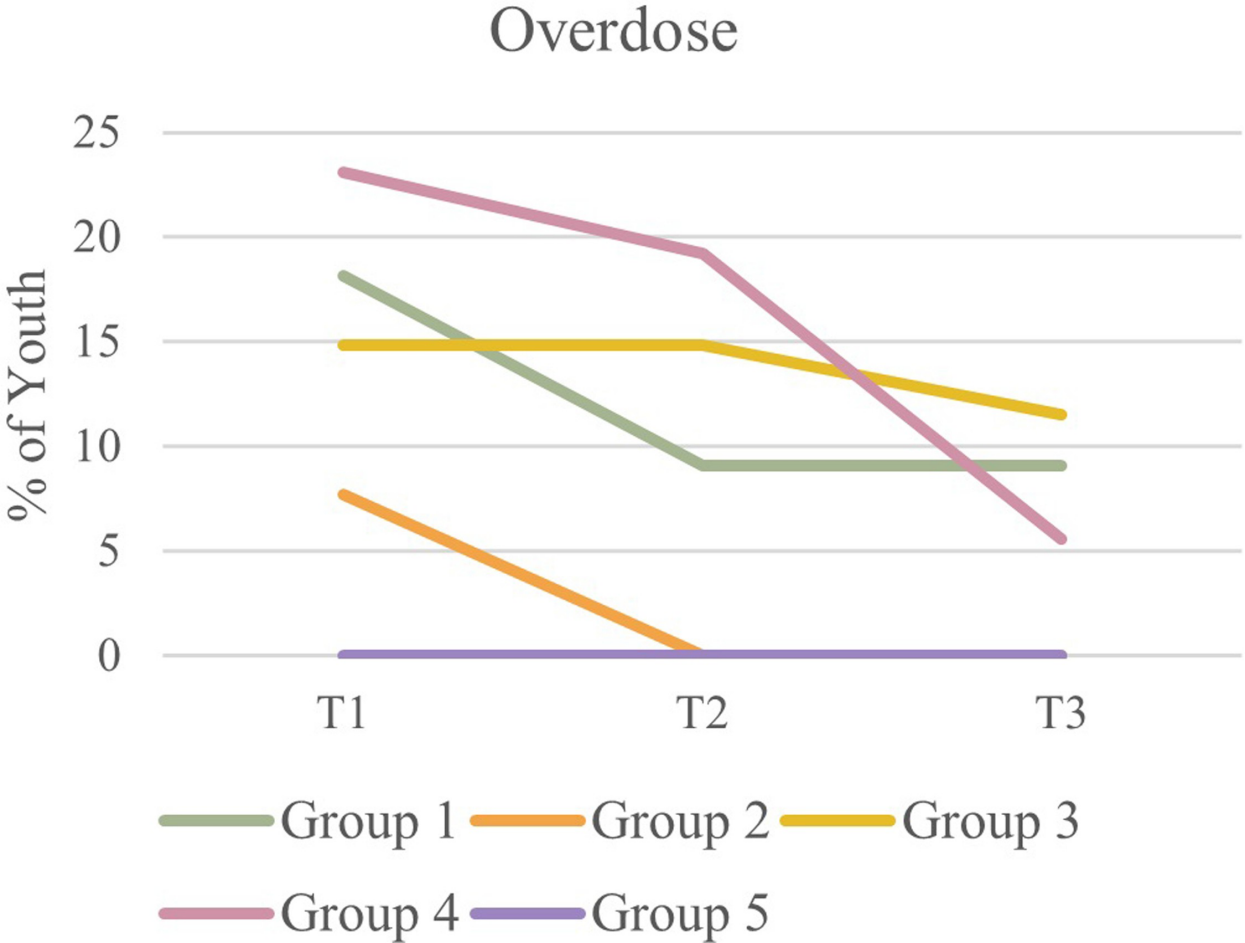
Overdose among youth over time.

McNemar’s tests determined that there were no statistical differences in the proportion of youth engaged in problematic substance use across timepoints. See Fig 6 for a graphical representation of substance use among participants over time. McNemar’s tests also did not indicate any statistical differences in youth reporting having experienced a recent alcohol or drug overdose between T1 and T2 or T1 and T3. See Fig 7 for a graphical representation of overdose among youth over the course of the pandemic.

### Mental health care access

**Fig 8:**
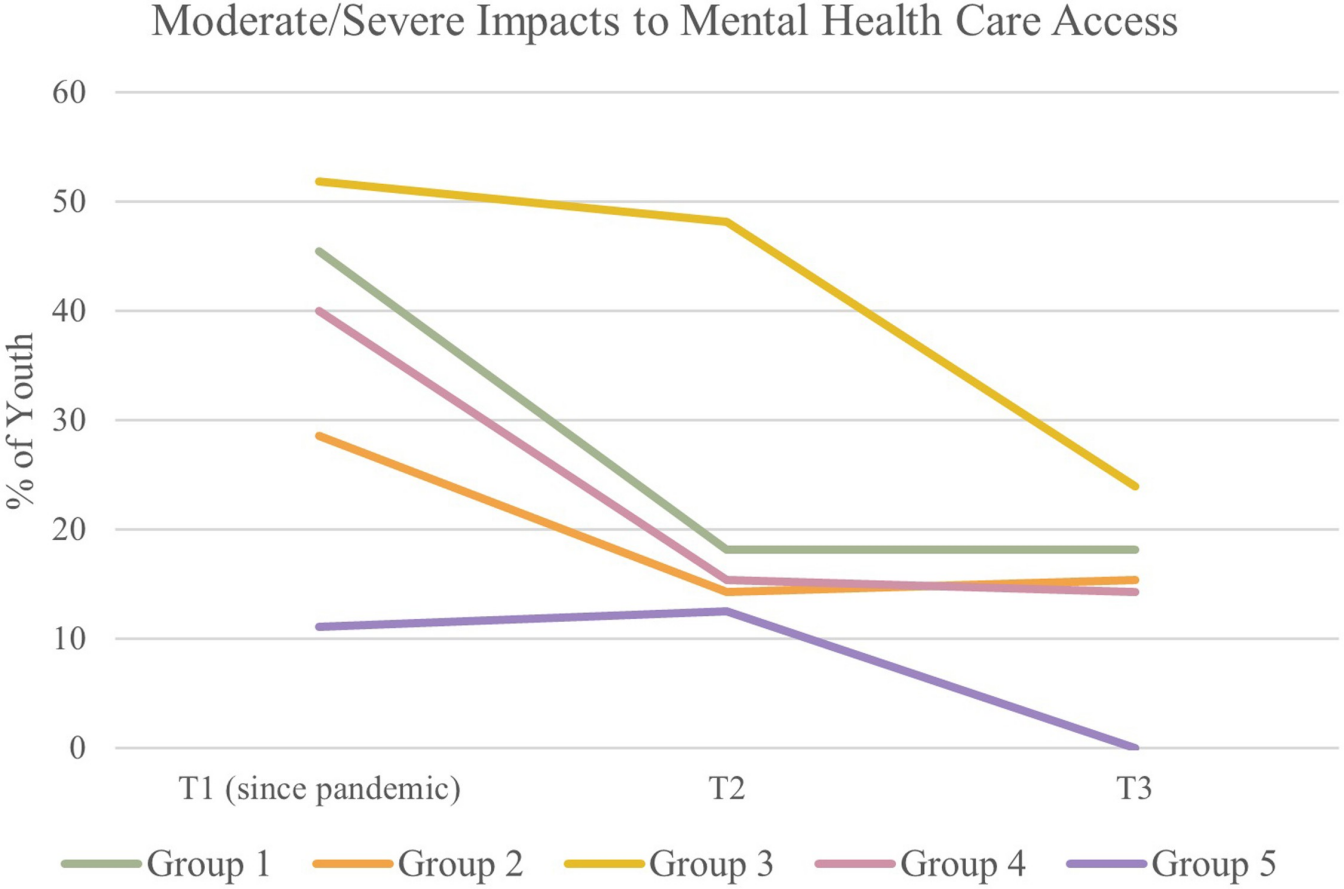
Access to mental health care over time.

Friedman Rank Sum tests indicated a statistical difference in access to mental health care across timepoints in Group 5 (p = 0.0160). In Group 5, a post-hoc Wilcoxon signed rank test demonstrated a significant difference in mental health care between T1 and T3 (p = 0.0310) but not between T1 and T2 (p = 0.5827). This change was characterized mostly by youth who reported experiencing changes to their mental health care at T1 but reported no changes or not requiring/attempting to access mental health care at T3. See Fig 8 for a graphical representation of the impacts to mental health care access over time.

## Discussion

In this study we aimed to understand how mental health changed among 2SLGBTQ+ youth at risk of, and experiencing, homelessness throughout the COVID-19 pandemic. Our findings illustrate that 2SLGBTQ+ youth experienced significant mental health challenges during the pandemic, overall, there were high rates of severe anxiety and depression among approximately half of participants, varying slightly across data collection timepoints. These data are consistent with existing literature reporting high rates of mental health challenges among 2SLGBTQ+ youth experiencing homelessness prior to the COVID-19 pandemic, [10, 14] as well as recent research reporting increased mental health challenges among 2SLGBTQ+ during the COVID-19 pandemic [8, 9, 15–17].

Overall, our findings illustrate that 2SLGBTQ+ youths’ mental health remained stable, although poor, over the course of 2021, consistent with existing findings among youth more broadly [22] Our findings suggest that the mental health among 2SLGBTQ+ youth experiencing homelessness may have been affected during the initial phase and lockdowns of the COVID-19 pandemic. Even though we found that participants’ mental health improved throughout the COVID-19 pandemic, specifically during our data collection period, 2SLGBTQ+ youth continue to experience poor mental health. Similar to our findings, other studies have found a decrease in mental health among individuals at the early stages of the COVID-19 pandemic and improved throughout [28]. However, research has reported that many people struggle to readjust to the ‘new normal’ and those with pre-existing mental health challenges were disproportionately affected [40]. Numerous mental health services have also been impacted by the COVID-19 pandemic, resulting in the majority of participants reporting unmet mental health needs and limited access to mental health care. This is similar to previous literature that has reported unmet mental health needs among young adults throughout the COVID-19 pandemic [41, 42].

We cannot view the pandemic as a single event, but rather a fluid event, given the various periods of lockdowns and restrictions, which undoubtedly impacted participants in a variety of ways. The sample was divided into five groups based on the time of recruitment/baseline survey completion. These five groups help account for some of the variability due to the different waves of the COVID-19 pandemic. There were no significant changes observed for groups 1 to 3 for all of the measures. Group 4 showed significant improvements in depression, anxiety, substance use, overdose, NSSI, and suicidality, and other groups showed non-significant but similar trends. For example, group 5 also showed improvements in depression, anxiety, substance use, overdose, NSSI, and suicidality. However, these improvements were most evident in Group 4, the subsample who completed the survey beginning in April 2021, with follow-up surveys in July 2021 and October 2021 (i.e., later on in the pandemic). This distinction is likely due to multiple factors, potentially including the timing of COVID-19 waves and related health and safety restrictions in Ontario. In April 2021, Ontario was in a strict lockdown during the third wave of COVID-19, with cases decreasing and restrictions lifting by June 2021.

The mental health among 2SLGBTQ+ youth may have improved as a result of increased access to resources and social supports and decreased risk of COVID-19 infection over the summer and early fall of 2021. This is reflected in our results as all groups reported decreases in impacts to mental health care over the three timepoints. Several other studies have reported improvements in mental health during months of warmer weather and lower infection rates throughout the pandemic;[27, 28] however, some studies conversely show lower mental health symptoms during lockdown phases compared to prolonged phases of the pandemic [26]. Additionally, our analyses showed that the living situations of youth in Group 4 changed significantly over time, characterized by youth who were living in public spaces or at a (non-partner, non-parent) friend/family member’s place in April 2021 but transitioned into different living situations by October 2021. Moving into a safer and/or less precarious living situation may also have contributed to the improvements in mental health seen in this group, as supported by several findings regarding the impact of housing and family functioning on youth mental health during the pandemic [22, 24, 27, 43]. There were no differences in mental health outcomes between groups based on gender identity, sexual orientation, or race, though the groups may have differed in other demographic characteristics that could impact mental health, resilience, and access to supports.

Our study has several limitations. The key limitation is that 2SLGBTQ+ youth at-risk of, and experiencing, homelessness has been found to experience high rates of mental health issues and housing precarity prior to the COVID-19 pandemic, which makes it difficult to attribute the results directly to the effects of the pandemic. Second, although we worked with a wide range of organizations and community partners to recruit a diverse and representative sample of 2SLGBTQ+youth, we experienced challenges with participant recruitment. Likewise, our sample contains many youth who were connected to services and have access to technology. Therefore, this sample may have excluded some of the most marginalized 2SLGBTQ+youth. Therefore, we are unable to determine the extent to which our sample reflects the broader population of 2SLGBTQ+youth at risk of, and experiencing, homelessness during the COVID-19 pandemic. Our sample was also too small to conduct analyses of different experiences by gender identity and sexual orientation, along with other social identities including ethno-racial identity.

## Conclusion

The mental health among 2SLGBTQ+ youth participants worsened during the initial wave of the COVID-19 pandemic but improved marginally throughout. However, 2SLGBTQ+ youth experiencing homelessness continue to experience mental health concerns, including high rates of depression and anxiety. This study highlights the need for 2SLGBTQ+ inclusive and affirming mental health care services to address the social and mental health issues that have been exacerbated by the COVID-19 pandemic.

## Supporting information

S1 Checklist (DOCX)
